# Supplementary material for: The ATP-dependent chromatin remodelling enzyme Uls1 prevents Topoisomerase II poisoning
Source: Nucleic Acids Res. 2019 May 20;47(12):6172–83. doi: 10.1093/nar/gkz362 (PMC6614809; doi:10.1093/nar/gkz362)
Supplement: gkz362_Supplemental_Files [file gkz362_supplemental_files.pdf]

Supplementary Figure S1

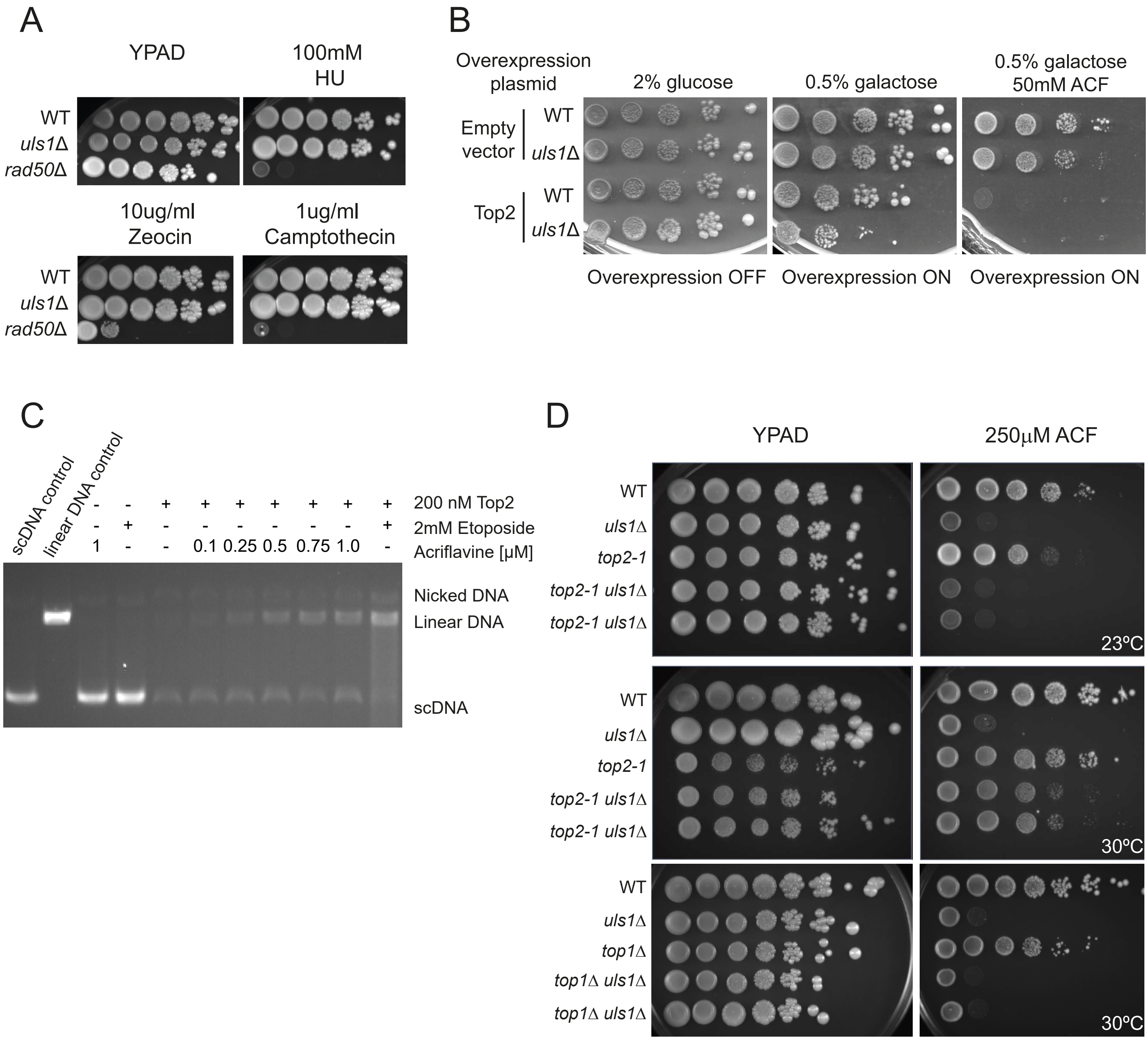

Supplementary Figure S2

A

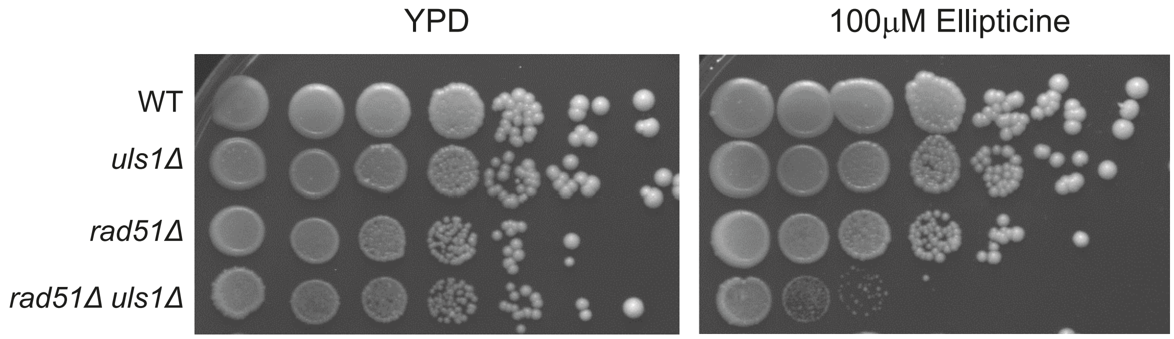

B

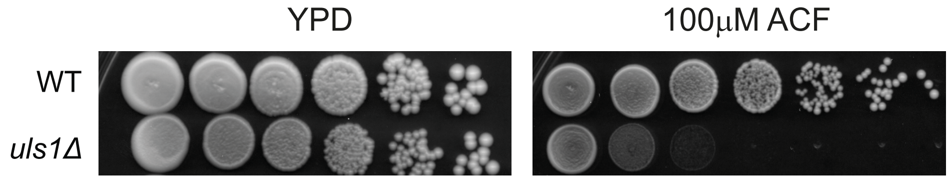

C

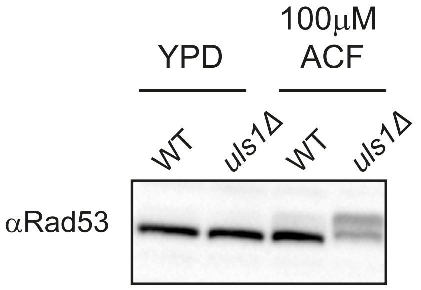

Supplementary Figure S3

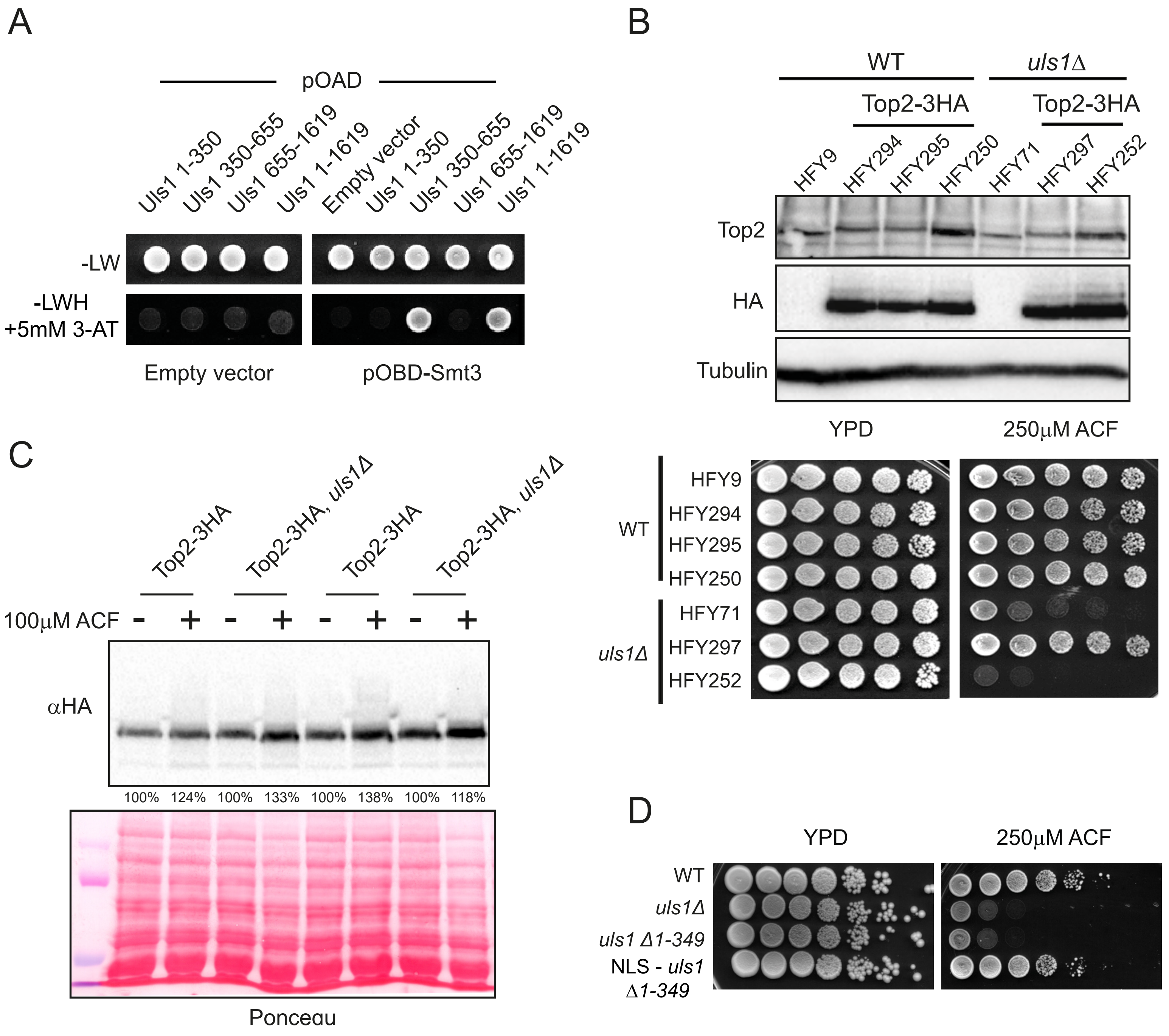

Supplementary Figure S4

A

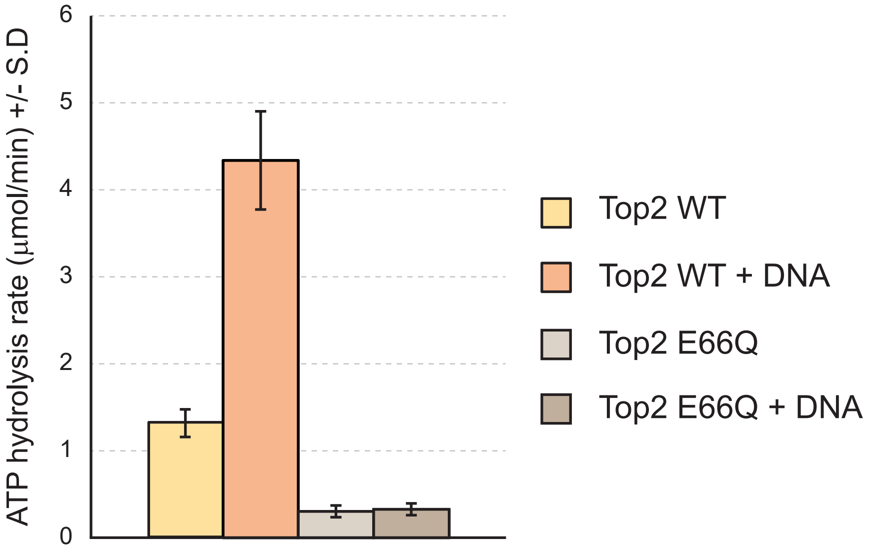

B

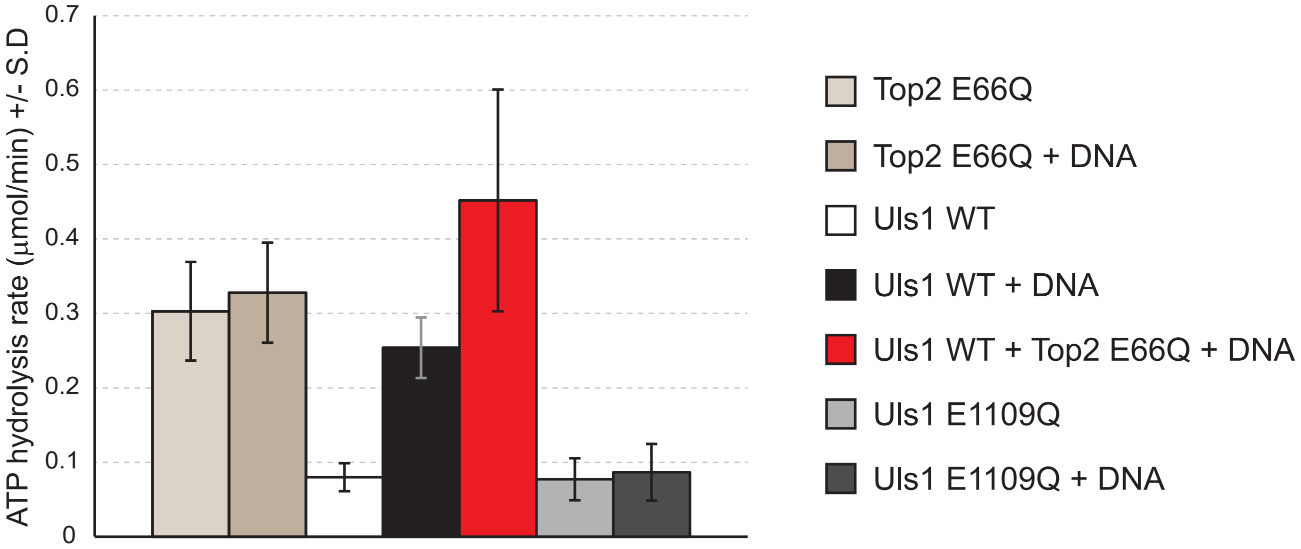

Supplementary Figure S5

A

Annotation	Number of peaks in condition			
	Top2 YPD WT	Top2 YPD *uls1Δ*	Top2 ACF WT	Top2 ACF *uls1Δ*
Gene	435	253	948	1650
Other	115	74	180	384
tRNA	74	47	42	71
ARS	4	3	5	14
Total peaks	628	377	1175	2120

B

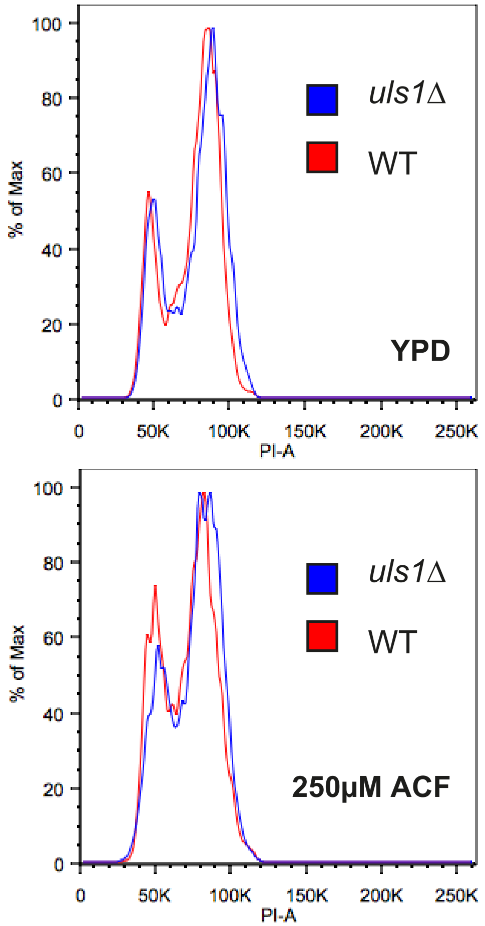

C

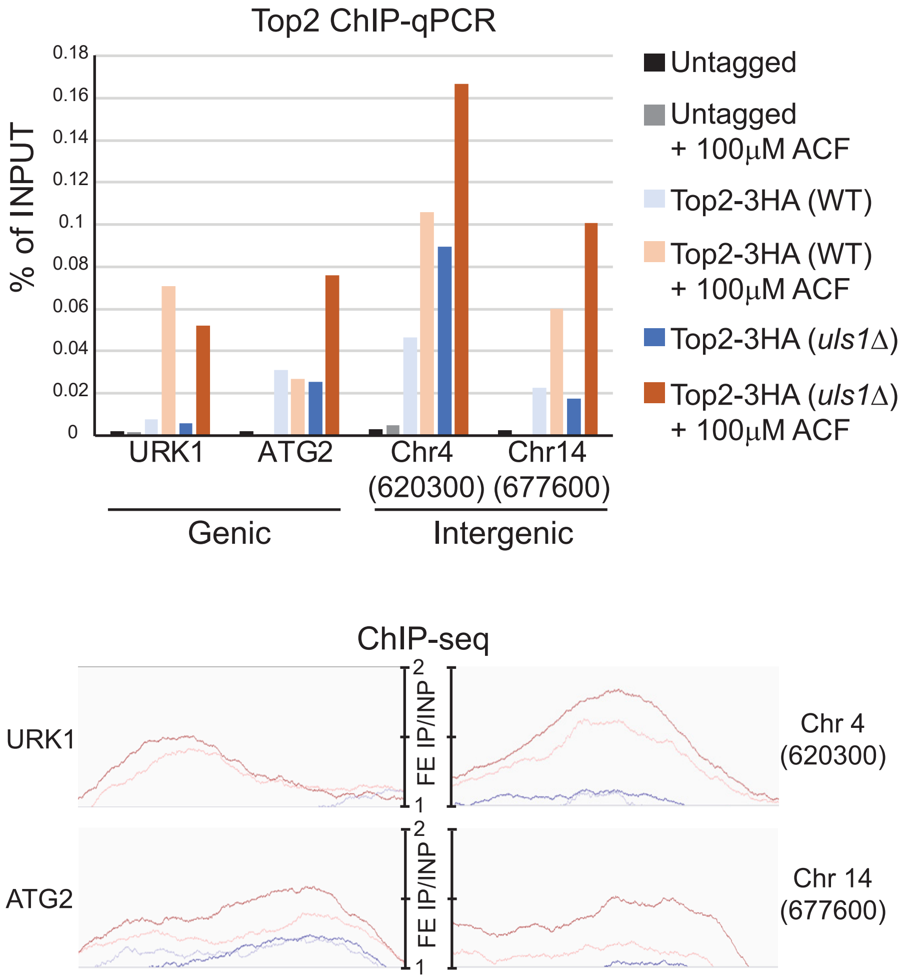

D

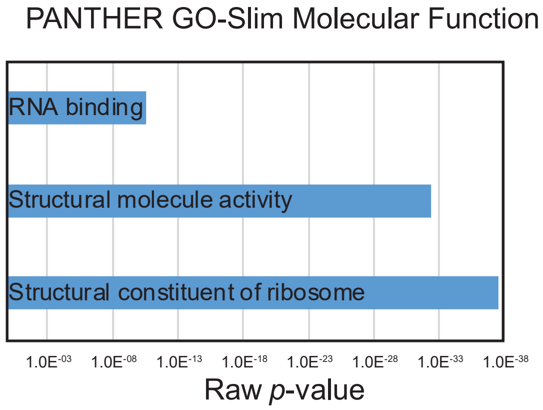

Supplementary Figure S6

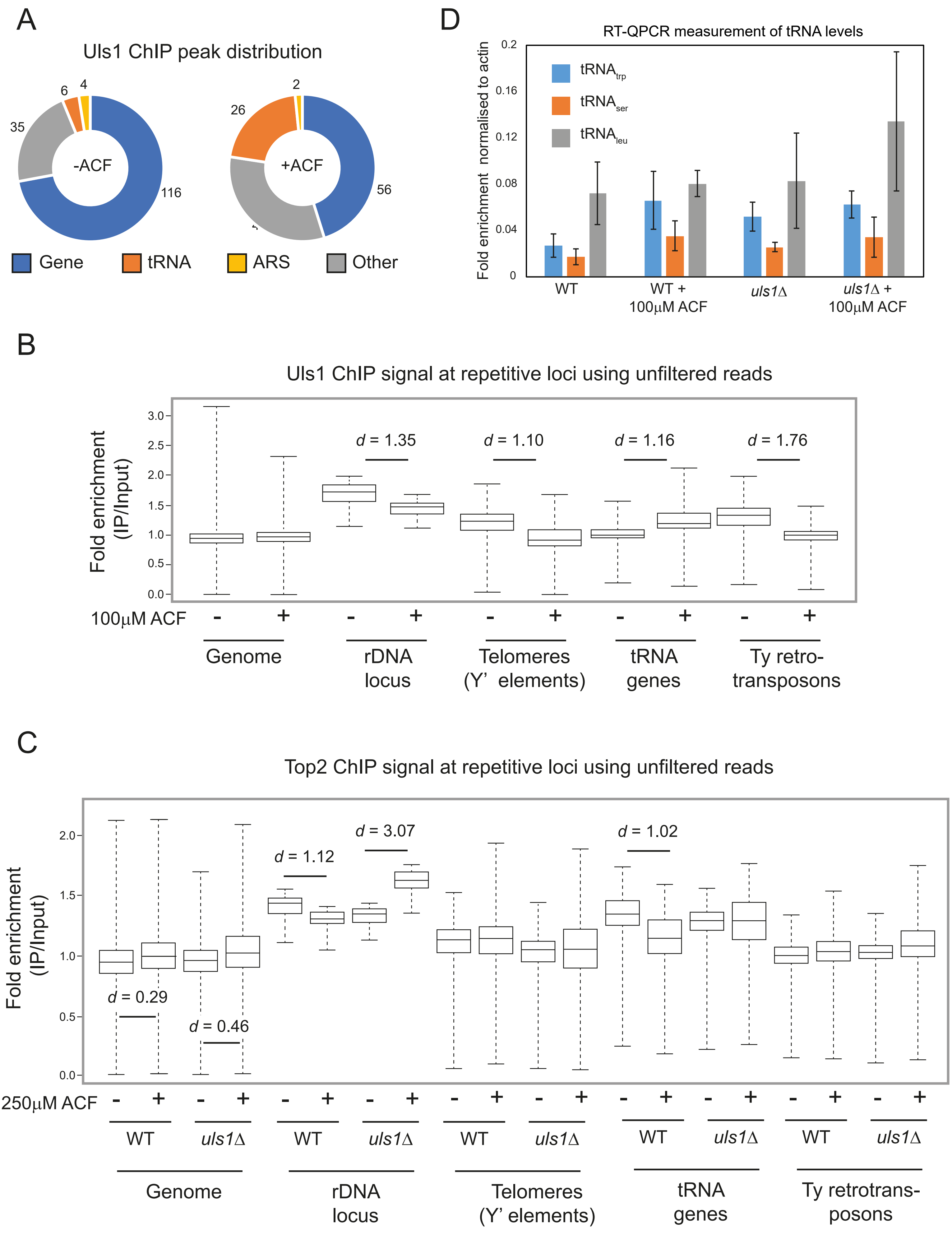

Supplementary Figure S1. Acriflavine is a Top2 poison. (**A**) Deletion of *ULS1* does not cause sensitivity to the replication inhibitor, Hydroxyurea (HU), the DNA SSB and DSB forming drug Zeocin or the Top1 poison Camptothecin. (**B**) 10-fold serial dilution of yeast containing either an empty vector or a vector driving expression of Top2 (HFY185) under control of the *GAL1* promoter. Overexpression of Top2 is toxic to *uls1*Δ cells and is synergistically lethal with ACF. (**C**) *in vitro* cleavage assay. 200nM supercoiled pUC18 plasmid DNA (scDNA) was incubated for 30mins at 30°C with the indicated amounts of Top2 and either etoposide or ACF. Addition of ACF induced DNA cleavage, seen by the appearance of linear DNA, at lower concentrations than the positive control Top2 poison, etoposide. (**D**) *uls1*Δ ACF sensitivity is suppressed the *top2-1* hypomorph at the semi-permissive temperature (30°C) but not at the permissive temperature (23°C). Additionally, deletion of *TOP1* does not result in ACF sensitivity. These data re-enforce the point that ACF induced lethality in *uls1*Δ strains is specifically due to Top2 as it can be suppressed by reducing Top2 protein level but not by deleting Top1.

Supplementary Figure S2. Deletion of *ULS1* sensitises yeast to ellipticine and ACF activates the DNA damage checkpoint. (**A**) 10-fold serial dilution of yeast showing that *ULS1* deletion causes sensitivity to the Top2 poison Ellipticine but only in a sensitising background. A *uls1*Δ*, rad51*Δ double mutant strain (HFY33) is significantly more sensitive than a single *rad51*Δ strain (HFY27). (**B**) 100μM ACF is toxic to *uls1*Δ cells. (**C**) 100μM ACF is sufficient to induce activation of the DNA damage checkpoint in *uls1*Δ yeast as visualised by Rad53 phospo-shift using an anti-Rad53 antibody (Abcam 104232).

Supplementary Figure S3. Deletion of ULS1 does not alter Top2 protein levels. (**A**) Yeast 2-hybrid assay showing that full-length Uls1 (HFP136) and Uls1 35-655 (HFP133) can interact with Smt3 (yeast SUMO) *in vivo* (HFP288). (**B**) Top panel shows Top2 protein levels as measured by Western blot using anti-Top2 (TopoGEN TG2014), or anti-HA (Roche ROAHAHA) antibodies with an anti-Tubulin (Sigma T5168) loading control. Top2 protein levels are comparable between congenic wildtype and *uls1*Δ yeast (HFY9 with HFY71, HFY294 and HFY295 with HFY297 and HFY250 with HFY252). The bottom panel illustrates that HA tagging the endogenous TOP2 locus (HFY297) suppresses ACF sensitivity in contrast to introducing an extra HA-tagged copy of TOP2 (HFY252). (**C**) Two independent biological replicates looking at Top2 protein levels in WT (HFY250) and *uls1*Δ (HFY252) in the presence and absence of ACF. Signal intensity was quantified using ImageJ and the numbers below each lane display Top2 protein intensity normalised to amount in the absence of ACF. There is a mild increase in the level of Top2 when ACF is added. However, this increase is not markedly different between WT and *uls1*Δ strains and is therefore unlikely to explain the dramatically different phenotype of WT and *uls1*Δ yeast exposed to ACF (**D**) 10-fold serial dilutions of the indicated genotypes showing that Uls1 needs to be nuclear for its function and that the first 349 amino acids contain a nuclear localisation sequence (NLS). *uls1*Δ *1-349* (HFY234) phenocopies *uls1*Δ (HFY71). However, its function is fully rescued by addition of an SV40 NLS (HFY281).

Supplementary Figure S4. Top2 does not stimulate Uls1's ATPase activity. (**A**) ATP hydrolysis rates for the indicated proteins. The graph shows the average +/- the standard deviation of three independent experiments. 50nM wildtype Top2 (HFP185) or the ATPase dead E66Q mutant (HFP271) was incubated with or without 100μM salmon sperm DNA. (**B**) 15nM Uls1 (HFP350) and/or 50nM Top2 E66Q (HFP271) was incubated with or without 100μM salmon sperm DNA. Uls1 has weak DNA-stimulated ATPase activity which is not significantly further stimulated by Top2. Top2 E66Q was used to preferentially monitor the ATPase activity of Uls1.

Supplementary Figure S5. Top2 peak number increases in the presence of ACF. (**A**) Table showing the number of Top2 peaks associated with RNA Pol II genes, tRNA genes and replication origins (ARS) in WT (HFY250) or *uls1*Δ (HFY252) cells in the presence (ACF) or absence (YPD) of ACF. (**B**) The cell cycle profiles of WT and *uls1*Δ cells in the absence of ACF are indistinguishable by FACS. In the presence of ACF, there are fewer *uls1*Δ cells in G1 phase compared to WT, consistent with mild DNA-damage induced G2/M arrest. It suggests that differences in cell cycle profile are not driving the observed changes in Top2 chromatin binding. (**C**) ChIP qPCR (top panel) and ChIP-seq (bottom panel) at four different regions display the same overall trends +/- ACF. Top2 ChIP qPCR was performed on WT (HFY294) and *uls1*Δ cells (HFY297) where there is only one copy of TOP2 and this is HA tagged. (**D**) Gene ontology analysis of regions that show a decrease in chromatin-bound Top2 after the addition of ACF in wildtype cells. Ribosomal protein genes are significantly enriched.

Supplementary Figure S6. Analysis of Top2 and Uls1 ChIP signal at repetitive loci. (**A**) Graph showing the number of Uls1 peaks associated with RNA Pol II genes, tRNA genes and replication origins (ARS) in WT (HF176) cells in the presence (ACF) or absence (YPD) of ACF. (**B**) Pairwise comparison of the average Uls1 ChIP enrichment using unfiltered reads across the genome and specifically within the rDNA locus, telomeric Y' elements, tRNA genes and Ty retrotransposons +/- 100μM ACF. All pairwise comparisons (+/- ACF) with a Cohen's d value > 0.2 are displayed. (**C**) Pairwise comparison of the average Top2 ChIP enrichment using unfiltered reads across the genome and specifically within the rDNA locus, telomeric Y' elements, tRNA genes and Ty retrotransposons +/- 250μM ACF in either WT (HFY250) or *uls1*Δ (HFY252) cells. All pairwise comparisons (+/- ACF) with a Cohen's d value > 0.2 are displayed. (**D**) RT-qPCR to measure tRNA levels. RNA was extracted from yeast using hot phenol RT reaction carried out using Promega ImProm-II kit. Three groups of yeast tRNA genes quantified using primers described in Chen and Gartenberg (Genes & Dev. 2014). No DNA was detected in qPCR when reverse transcriptase was left out of reaction. We do not see any statistically significant differences in tRNAser, tRNAtrp or tRNAleu expression in either WT or *uls1*Δ.cells +/- ACF. The graph plots the average +/- the standard deviation of at least three independent experiments normalised to actin mRNA.

Supplementary Methods

ChIP seq library preparation

Due to the low quantity of DNA present after immunoprecipitation, to ensure there is enough sample DNA for amplification and sequencing two experimental replicates are combined before ChIP-seq library preparation.

Step 1 of library preparation allows repair of DNA ends. To 80µl pooled DNA, add 20µl MMX1 (1x T4-ligase buffer with ATP, 0.4mM dNTPs, 15 units T4 DNA polymerase, 10 units Klenow DNA polymerase, 30 units T4 polynucleotide kinase) and incubate for 60 minutes at 20°C. DNA is purified by addition of a 1:1 v/v of AMPure XP magnetic beads, incubating for 5 minutes before washing twice with 200µl 70% EtOH using a magnetic rack. Residual EtOH is removed before elution of DNA in 41µl H2O.

Step 2 of library preparation adds an additional adenine nucleotide to DNA ends to which adapters will later be ligated. To 41µl DNA, add 9µl MMX2 (1x Klenow buffer, 2mM dATP, 15 units Klenow exo-), incubating for 30 minutes at 37°C. DNA is purified as in step 1 using a 1:1 v/v of Agencourt AMPure XP magnetic beads, eluting in a final volume of 20µl H2O.

Step 3 of library preparation ligates adapters onto DNA which are used later as a template for PCR amplification of ChIPed DNA fragments to generate the final tagged DNA library. Adapter is ordered as two oligonucleotides (HFO424/425, Illumina paired end adapter sequence). HFO425 is phosphorylated as per manual specifications using T4 polynucleotide kinase (Thermo Fisher EK0032) before annealing to HFO424 by mixing an equimolar ratio of the two oligos and heating to 95°C for 5 minutes, reducing the temperature by 5°C every 5 minutes until reaching 25°C. Adapter is then run into a 1% agarose gel, gel purified and stored at -20°C before use. To ligate adapter to DNA, to 20µl DNA add 30µl MMX3 (8nM adapter, 1x T4 DNA ligase buffer with ATP, 400 units T4 DNA ligase), incubating overnight at room temperature. DNA is purified as in step 1 with a 1:1 v/v of Agencourt AMPure XP magnetic beads, eluting in 20µl H2O.

Step 4 of library preparation amplifies the DNA samples using primers complimentary to the adapters now ligated onto each DNA molecule, with each primer also containing an additional unique sequence

tag which is used to identify each sample after sequencing of the DNA. Details of the primers used can be found in Table 6–6. For each sample, 3 PCR reactions are set up containing 3µl DNA and 47µl MMX4 (1X HF buffer, 3 units Phusion DNA polymerase, 0.3µM oligos (HFO426 and sequencing primer), 0.4µM dNTPs, 200mM Trehalose). The PCR is carried out by denaturing for 3 mins at 98°C before 16-20 cycles of amplification (98°C 15 secs, 60°C 25 secs, 68°C 1 min) and a final step at 68°C for 5 mins. The three PCRs are then pooled and ran on a TAE gel containing 1% agarose and 0.5µg/ml EtBr. Once the gel has ran long enough to separate free adapter from amplified DNA, bands are cut from the gel at a size of 600bp and under, avoiding contamination with adapter. DNA is purified from the gel using Quaigen MiniElute columns (Quaigen, 28006) as per kit specifications except gel was melted at 37°C, eluting from the column in 10µl EB.

DNA concentration was quantified via Qubit using 1:199 DNA:Qubit dsDNA High Sensitivity assay working solution (Invitrogen, Q32854, as per kit specifications). DNA quality and mean fragment size were checked on a TapeStation, running samples onto D1000 ScreenTape with D1000 reagents (Agilent, 5067-5582, 5067-5583).

ChIP-seq bioinformatic analysis

Building the W303 genome annotation

A whole-genome sequence for the W303 strain of S. cerevisiae was published by Matheson et al. (2017), containing 16 nuclear chromosomes, the mitochondrial genome and additional plasmids present in the sequenced strain. We were unable to obtain a copy of the Matheson genome annotation so generated our own. Initial annotation was carried out by inputting the W303_LYZE genome sequence (Genbank GCA_002163515.1, Assembly name ASM216351v1) into the Yeast Genome Annotation Pipeline (YGAP, Proux-Wéra et al., 2012). We used this as a basis for our genome annotation, using BLASTn (Altschul et al., 1990) to identify any ORFs which were not annotated by YGAP. YGAP identified 5634 ORFs, with 6600 present in the S288C reference, the gold standard of genome references in yeast (Saccharomyces Genome Database, version R64-2-1, Engel et al., 2013). 141 tRNA genes were identified, with 299 being present in the S288C reference. Where an unidentified gene had high similarity to Y prime helicase elements, this was labelled as “Y’ element” (54 items), and where it had high similarity to gag pol genes, this was labelled as “TKP/TY” (86 items). Any elements

that could not be identified were labelled as "unknown" (4/5634 ORFs). Gene lengths were checked against S288C and a note was made for each gene as to whether they were the same or not, with 524/5634 differing in size in W303 compared to S288C. Additional information for each ORF was extracted from the YGP S288C reference using excel, including gene names, aliases and gene ontology information. ARS were identified using Biopython BioSeqIO (Cock et al., 2009), searching for variants of the consensus sequence for ARS (ATTTATATTTA, TTTTATATTTA, ATTTATGTTTA, TTTTATGTTTA, ATTTATATTTT, TTTTATATTTT, ATTTATGTTTT, TTTTATGTTTT), finding 333 ARS. Centromeric sequences were identified by blasting the nucleotide sequence of the S288C CEN1-16. CEN5 was identified by blasting for conserved centromeric elements CDEI and CDEIII as it had little homology to S288C CEN5.

Read mapping and peak calling

Sequenced fastq.gz were catenated and renamed (ULS1_YPD_WT, ULS1_ACF_WT, TOP2_YPD_WT, TOP2_ACF_WT, TOP2_YPD_ULS1, TOP2_ACF_ULS1). Empty adapter was trimmed from sequencing files using cutadapt (Martin, 2011). Data quality was checked using FASTQC (Andrews, 2010). Reads were mapped to the *S. cerevisiae* W303 genome (Matheson et al., 2017) using BWA (Li and Durbin, 2010) with default parameters for paired end sequences to generate read alignment files in SAM format. This was then converted to BAM format using samtools view, sorted using samtools sort, and indexed using samtools index, all with default parameters (Li et al., 2009). Indexed BAM files were quality filtered using samtools view (Li et al., 2009) with a quality filter of 28 and a -F FLAG value of 1796. QualiMap (Okonechnikov et al., 2016) was used to check the quality of alignment in both the unfiltered and quality filtered BAM files. Replicate correlation was checked using Pearson's correlation, calculated using deepTools multiBamSummary and plotCorrelation (Ramírez et al., 2016). Replicates or inputs (within one genotype) were pooled using samtools merge with default parameters. The resulting file was then sorted using samtools sort and indexed using samtools index, all with default parameters (Li et al., 2009).

Peak calling was carried out using MACS2 subcommands (Zhang et al., 2008). PCR duplicates were filtered using macs2 filterdup. IP pileup track was generated using macs2 pileup, extending reads to the average fragment size. INP local lambda track was generated using macs2 pileup (-B), generating three tracks where reads were extended in both directions by half the average fragment size

(termed “d”), 500bp (termed “1kb_slocal”), or 2500bp (termed “5kb_llocal”). 1kb_slocal and 5kb_llocal were normalised to “d” sized fragments using macs2 bdgopt (-m multiply). All tracks were combined to generate “local lambda” background track using macs2 bdgcmp (-m max), also normalising to maximum background noise (number of reads in INP x average fragment length / genome size) using macs2 bdgopt (-m max). IP and local lambda were normalised to counts per million (CPM) using macs2 bdgopt (-m multiply). A p-value statistical track was generated using macs2 bdgcmp (-m ppois). Peak calling was carried out using macs2 bdgpeakcall with a p-value cut-off of 0.1. IP/INP fold enrichment tracks were generated using macs2 bdgcmp.

Differential analysis

Differential analysis compares signal at peak regions between different datasets. To do this, the peaks called in all Top2/Uls1 datasets must be combined to give a list of the regions at which we will be completing differential analysis. This is completed using bedtools intersectBed and mergeBed (Quinlan and Hall, 2010).

For example, for Uls1 ChIP the following commands were used:

```
intersectBed -a ${ULS1_YPD_WT} -b ${ULS1_ACF_WT} -u | sort -k1,1 -k2,2n -k3,3n >
common_peak1.bed
intersectBed -a ${ULS1_ACF_WT} -b ${ULS1_YPD_WT} -u | sort -k1,1 -k2,2n -k3,3n >
common_peak2.bed
intersectBed -a ${ULS1_YPD_WT} -b ${ULS1_ACF_WT} -v | sort -k1,1 -k2,2n -k3,3n >
unique_${ULS1_YPD_WT}.bed
intersectBed -a ${ULS1_ACF_WT} -b ${ULS1_YPD_WT} -v | sort -k1,1 -k2,2n -k3,3n >
unique_${ULS1_ACF_WT}.bed
cat common_peak1.bed common_peak2.bed unique_${ULS1_YPD_WT}.bed
unique_${ULS1_ACF_WT}.bed > temp_all_peaks.bed
sort -k1,1 -k2,2n -k3,3n temp_all_peaks.bed > all_peaks.bed
mergeBed -i all_peaks.bed > all_peaks_merged.bed
```

A bedgraph file is then generated containing signal only at the peak regions (all_peaks_merged.bed) using bedtools intersectBed (-wb) (Quinlan and Hall, 2010).

A boxplot of all coverage and peak signal is then plotted using an R script and datasets compared pairwise for the variable Cohen's D (Cohen, 1988) to see whether the datasets are statistically different, and if so whether any differences are small (>0.2), medium (>0.5) or large (>0.8). This used the following code:

```
library(lsr)
#Describing the variables
ALLCOVERAGE1 <- read.table("sample1_coverage.bdg")
ALLCOVERAGE2 <- read.table("sample2_coverage.bdg")
PEAK1 <- read.table("sample1_coverage_peakregions.bdg")
PEAK2 <- read.table("sample2_coverage_peakregions.bdg")
#Is data normally distributed?
dALLCOVERAGE1 <- density(ALLCOVERAGE1$V4)
dALLCOVERAGE2 <- density(ALLCOVERAGE2$V4)
dPEAK1 <- density(PEAK1$V4)
dPEAK2 <- density(PEAK2$V4) png("allcoveragesample1_density.png")
plot(dALLCOVERAGE1)
dev.off() png("allcoveragesample2_density.png") plot(dALLCOVERAGE2)
dev.off()
png("peakcoveragesample1_density.png")
plot(dPEAK1)
dev.off()
png("peakcoveragesample2_density.png")
plot(dPEAK2)
dev.off()
#Generate box plots of pairwise data
png("boxplot.png")
```

```
boxplot(ALLCOVERAGE1$V4, ALLCOVERAGE2$V4, PEAK1$V4, PEAK2$V4, range=0,
names=c("all_s1", "all_s2", "peaks_s1", "peaks_s2"))
dev.off()
#Cohens D
sink(cohensD')
cat("====================\n")
cat("cohens D\n")
cat("====================\n")
cat("ALLCOVERAGE\n")
cohensD(ALLCOVERAGE1$V4, ALLCOVERAGE2$V4)
cat("PEAKREGIONS\n")
cohensD(PEAK1$V4, PEAK2$V4)
sink()
```

Peak annotation

Peaks were annotated to our W303 annotation using bedtools closest (Quinlan and Hall, 2010).

Transcription level analysis

Genic peaks were annotated with their gene transcription level by comparing to a published dataset for S288C where RNA-seq was carried out on cells before and after exposure to hypoxia. We used the dataset at the 0 hour timepoint where cells had been grown to midlog phase (Bendjilali et al., 2016). Within this dataset we discarded all data where 0 reads mapped and selected for only chromosomal ORFS (not within the 2μ plasmid/mitochondrial genome or corresponding to special RNA structures which had no corresponding ORF). The RPKM values for the remaining genes were ranked from low to high and annotated as being the “bottom 20%” (1280 genes), “mid 60%” (3829 genes) or “top 20%” (1278 genes) in terms of transcription levels. Excel was then used to annotate each genic peak within a dataset with its transcription level using the OFFSET and MATCH functions.

Transcription start site analysis

Analysis was carried out using the R based packages rtracklayer, ChIPseeker and Genomic Features (Lawrence, 2013; Lawrence et al., 2009; R Core Team, 2018; Yu et al., 2015). The input files for this analysis were our MACS2 peak files.

Repetitive region analysis

This analysis uses unfiltered reads where reads which map to multiple regions have not been removed. This analysis is slightly difficult in that you cannot use signal height to define whether the ChIPed protein is binding or not as the more repetitive the sequence, the higher the signal will be. However, if a pairwise comparison of two datasets within a repetitive region is carried out and one dataset shows significantly higher enrichment than the other, then it can be logically assumed that there is enrichment of the ChIPed protein within the “higher” dataset.

Analysis was carried out at subtelomeric loci (+/- 5kb from each chromosome end), tRNA (as within genome reference), TKP/TY transposons (as within genome reference), Y’ elements (as within genome reference) and the rDNA locus. We identified a partial fragment of the rDNA locus within our W303 reference by using blastn (Altschul et al., 1990) to search the sequence for a single rDNA repeat from within chromosome 12 in the S288C reference (Engel et al., 2013), aligning to the sequence of chromosome 12 within our W303 reference. According to SGD (Stanford University, 2012), the sequence of a single rDNA repeat within the S288C reference is on Chromosome 12 at 459,797-468,931. The top hit from this blast search within our W303 reference is on Chromosome 12 at 478,018-478,813, containing the sequence of part of the 35S rDNA locus.

First, a .bed file was generated containing the coordinates of all the regions within one repetitive region, for instance the 141 tRNA genes within our W303 reference. This was made within excel and saved as a tab delimited file. Data tracks were then generated with signal extracted at each repetitive region using intersectBed (Quinlan and Hall, 2010) and a peak list containing the SUM score under each peak in each dataset using the following commands for each dataset:

```
intersectBed -wb -a ${DATASET1} -b ${TEST}.bed > ${DATASET1}_${TEST}_temp.bdg
awk 'BEGIN{OFS="\t"}{print $1, $2, $3, $4}' ${DATASET1}_${TEST}_temp.bdg >
${DATASET1}_${TEST}.bdg
```

awk 'BEGIN{OFS="\t"}{a[$5,"_",$6,"_",$7]+=$4}END{for(i in a){print i,a[i]}}' ${DATASET1}_${TEST}.bdg | sort -k1,1 -k2,2n > ${DATASET1}_${TEST}_coverage_SUMpeakregions.bdg

with SUM scores pasted into one file using the command:

paste -d "\t" ${DATASET1}_${TEST}_coverage_SUMpeakregions.bdg ${DATASET2}_${TEST}_coverage_SUMpeakregions.bdg ... > ${OUT_SUMpeak}

where:

DATASET1= The FE IP/INP track for a particular condition in .bed format

TEST= The repetitive region we are analysing (tRNA, rDNA, TEL+/-5kb, TKP/TY and Y' elements)

DATASET_TEST= A bedgraph track containing the signal only at the repetitive region of interest

DATASET1_TEST_coverage_SUMpeakregions.bdg= A file containing the sum score of signal at each repetitive region element for dataset one

OUT_SUMpeak= A file containing the sum score of signal at each repetitive region element for all datasets to be compared (e.g. all ULS1 ChIP datasets)

Top2 vs Uls1 comparative analysis

This analysis allows comparison of Top2 and Uls1 ChIP data at all regions defined as peaks in either Uls1 ChIP or Top2 ChIP.

First, a peak list for all Top2 and all Uls1 peaklists is generated by catenating peak files into two merged peaklists. Next, the Top2 and Uls1 peak lists were compared using intersectBed (Quinlan and Hall, 2010), with the following commands:

intersectBed -a ${PEAK1}.bed -b ${PEAK2}.bed -u | sort -k1,1 -k2,2n -k3,3n > common_peak1.bed

intersectBed -a ${PEAK2}.bed -b ${PEAK1}.bed -u | sort -k1,1 -k2,2n -k3,3n > common_peak2.bed

intersectBed -a ${PEAK1}.bed -b ${PEAK2}.bed -v | sort -k1,1 -k2,2n -k3,3n > unique_${PEAK1}_temp.bed

intersectBed -a ${PEAK2}.bed -b ${PEAK1}.bed -v | sort -k1,1 -k2,2n -k3,3n > unique_${PEAK2}_temp.bed

```
cat common_peak1.bed common_peak2.bed > Top2_Uls1_commonpeaks_temp.bed
210
sort -k1,1 -k2,2n -k3,3n Top2_Uls1_commonpeaks_temp.bed > Top2_Uls1_commonpeaks.bed
mergeBed -i Top2_Uls1_commonpeaks.bed > Top2_Uls1_commonpeaks_merged.bed
sort -k1,1 -k2,2n -k3,3n unique_${PEAK1}_temp.bed > unique_${PEAK1}.bed
sort -k1,1 -k2,2n -k3,3n unique_${PEAK2}_temp.bed > unique_${PEAK2}.bed
```

where:

PEAK#= Merged peak file for ChIP to be compared (e.g. all Top2 peaks, or all Uls1 peaks)

Top2_Uls1_commonpeaks.bed= A peak list for locations where Top2 and Uls1 colocalise

Top2_Uls1_commonpeaks_merged.bed= A peak list for locations where Top2 and Uls1 colocalise where overlapping peaks have been merged (this removes all duplicate peaks where a peak was in both the Top2 and Uls1 dataset)

unique_PEAK#= A peak list for locations where only this ChIP factor localises (e.g. unique Top2 peaks, or unique Uls1 peaks)

Boxplots for the datasets are then generated using an R based script (R Core Team, 2018):

```
TOP2_YPD_WT_all <- read.table("../../coverage/TOP2_YPD_WT_coverage.bed")
TOP2_YPD_ULS1_all <- read.table("../../coverage/TOP2_YPD_ULS1_coverage.bed")
TOP2_ACF_WT_all <- read.table("../../coverage/TOP2_ACF_WT_coverage.bed")
TOP2_ACF_ULS1_all <- read.table("../../coverage/TOP2_ACF_ULS1_coverage.bed")
ULS1_YPD_WT_all <-read.table("../../coverage/ULS1_YPD_WT_coverage.bed") ULS1_ACF_WT_all
<-read.table("../../coverage/ULS1_ACF_WT_coverage.bed")
TOP2_YPD_WT_test <- read.table("TOP2_YPD_WT_unique_ULS1_peaks.bdg")
TOP2_YPD_ULS1_test <- read.table("TOP2_YPD_ULS1_unique_ULS1_peaks.bdg")
TOP2_ACF_WT_test <- read.table("TOP2_ACF_WT_unique_ULS1_peaks.bdg")
TOP2_ACF_ULS1_test <- read.table("TOP2_ACF_ULS1_unique_ULS1_peaks.bdg")
ULS1_YPD_WT_test <- read.table("ULS1_YPD_WT_unique_ULS1_peaks.bdg")
ULS1_ACF_WT_test <- read.table("ULS1_ACF_WT_unique_ULS1_peaks.bdg")
#Boxplots of data
```

```
png("boxplot.png")
par(mar=c(10.1,4.1,2.1,2.1))
boxplot(TOP2_YPD_WT_all$V4, TOP2_YPD_ULS1_all$V4, TOP2_ACF_WT_all$V4,
TOP2_ACF_ULS1_all$V4, ULS1_YPD_WT_all$V4, ULS1_ACF_WT_all$V4,
TOP2_YPD_WT_test$V4, TOP2_YPD_ULS1_test$V4, TOP2_ACF_WT_test$V4, TOP2_ACF_ULS1
_test$V4, ULS1_YPD_WT_test$V4, ULS1_ACF_WT_test$V4, range=0, names=c("TOP2_YPD_WT",
"TOP2_YPD_ULS1", "TOP2_ACF_WT", "TOP2_ACF_ULS1", "ULS1_YPD_WT", "ULS1_ACF_WT",
"TOP2_YPD_WT", "TOP2_YPD_ULS1", "TOP2_ACF_WT", "TOP2_ACF_ULS1", "ULS1_YPD_WT",
"ULS1_ACF_WT"), las=2)
dev.off()
```

Supplementary Table 1

Strain number (HFY)	Genotype	Details
9	W303*: ade2-1 trp1-1 his3-11 his3-15 ura3-1 leu2-3 leu2-112 can1-100, RAD5+*	WT
26	HFY9, *uls1::KAN*	*uls1Δ*
27	HFY9, *rad51::URA3*	*rad51Δ*
33	HFY27, *uls1::KAN*	*rad51Δ uls1Δ*
71	HFY9*, uls1::URA3*	*uls1Δ*
118	PJ69-4A*: trp1-901 leu2-3,112 ura3-52 his3-200 gal4Δ gal80Δ LYS2::GAL1-HIS3 GAL2-ADE2 met2::GAL7-lacZ*	Y2H strain
155	BCY123: MATa, Can1, ade2, trp1, Ura3-52, his3, leu2-3, 112, pep4::his+, prb1::leu2+, bar1::HisG+, lys2::pGAL1/10-GAL4+	Protein expression strain
176	HFY71*, uls1::FLAG-ULS1 - TRP1*	FLAG-Uls1
188	HFY9*, TOP2::NAT-TOP2*	clonNAT cassette was integrated ~200 bp upstream of TOP2 ORF
189	HFY71*, TOP2::NAT-TOP2*	clonNAT cassette was integrated ~200 bp upstream of TOP2 ORF, *uls1Δ*
225	HFY71, *uls1::uls1 Δ350-655 - TRP1*	FLAG-Uls1 Δ350-655 (Top2 interaction deletion)
230	HFY71, *uls1::FLAG-uls1 C1385S -TRP1*	FLAG-Uls1 C1385S (RING mutant)
234	HFY71, *uls1::FLAG-uls1 Δ1-349-TRP1*	FLAG-Uls1 Δ1-349
250	HFY188*, TOP2::TOP2-3xHA-KAN*	Top2-3xHA integrated adjacent to *TOP2*
252	HFY189*, TOP2::TOP2-3xHA-KAN*	Top2-3xHA integrated adjacent to *TOP2, uls1Δ*
261	HFY71, *uls1::FLAG-uls1 I7A, L9A, I371A, I372A, I373A, L374A, L470A, L473A, L525A, L526A, L527A, T528A, I543A, L544A -TRP1*	FLAG-Uls1 mutSIM
263	HFY71, *TOP2::top2 Y510C-KAN, uls1::URA3*	Top2 suppressor Y510C, *uls1Δ*

264	HFY71, *TOP2::top2 I1121V-KAN*	Top2 suppressor I1121V, *uls1Δ*
275	HFY71, *uls1::FLAG-uls1 E1109Q -TRP1*	FLAG-Uls1 E1109Q (ATPase mutant)
281	HFY71, *uls1::FLAG-NLS(SV40)-uls1 Δ1-349-TRP1*	FLAG-NLS-Uls1 Δ1-349
294	HFY9*, TOP2::TOP2-3xHA-KAN*	Top2-3xHA replaces *TOP2*
297	HFY71*, TOP2::TOP2-3xHA-KAN*	Top2-3xHA replaces *TOP2*, *uls1Δ*

Supplementary Table 2

Plasmid number (HFP)	Containing ORF	Type of vector
57	Empty	Gateway entry vector
122	Empty	pOAD expression vector
127	Empty	pOBD expression vector
133	Uls1 350-655	pOAD expression vector
134	Uls1 655-1619	pOAD expression vector
136	Uls1 1-1619	pOAD expression vector
150	Top2	pOBD expression vector
185	12URA-B-Top2(aa1-1428)	Top2 expression
193	Uls1 1-350	pOAD expression vector
219	pRSFDuet-1-6xHis-Twin-STEP-Uls1(aa350-655)	Uls1 expression (aa350-655)
221	pRSFDuet-1-6xHis-Twin-STEP-Uls1(aa655-963)	Uls1 expression (aa655-936)
222	pRSFDuet-1-6xHis-Twin-STEP-Uls1(aa1-350)	Uls1 expression (aa1-350)
271	12URA-B-Top2(aa1-1428, E66Q)	Top2 expression E66Q
273	12URA-B-Top2(aa1-1428, I1121V)	Top2 expression I1121V
288	Smt3	pOBD expression vector
362	Top2-3HA KanR	Top2-3HA tagging
385	TwinStrep-Uls1-6xHis	Uls1 expression (aa350-1619)
404	TwinStrep-Uls1-6xHis E1109Q	Uls1 expression ATPase null
